# Oxidation Resistance of the Sulfur Amino Acids: Methionine and Cysteine

**DOI:** 10.1155/2017/9584932

**Published:** 2017-12-27

**Authors:** Peng Bin, Ruilin Huang, Xihong Zhou

**Affiliations:** ^1^Key Laboratory of Agro-Ecological Processes in Subtropical Region, Institute of Subtropical Agriculture, Chinese Academy of Sciences, National Engineering Laboratory for Pollution Control and Waste Utilization in Livestock and Poultry Production, Changsha, Hunan 410125, China; ^2^University of Chinese Academy of Sciences, Beijing 10049, China

## Abstract

Sulfur amino acids are a kind of amino acids which contain sulfhydryl, and they play a crucial role in protein structure, metabolism, immunity, and oxidation. Our review demonstrates the oxidation resistance effect of methionine and cysteine, two of the most representative sulfur amino acids, and their metabolites. Methionine and cysteine are extremely sensitive to almost all forms of reactive oxygen species, which makes them antioxidative. Moreover, methionine and cysteine are precursors of S-adenosylmethionine, hydrogen sulfide, taurine, and glutathione. These products are reported to alleviate oxidant stress induced by various oxidants and protect the tissue from the damage. However, the deficiency and excess of methionine and cysteine in diet affect the normal growth of animals; thereby a new study about defining adequate levels of methionine and cysteine intake is important.

## 1. Introduction

**Figure 1:**
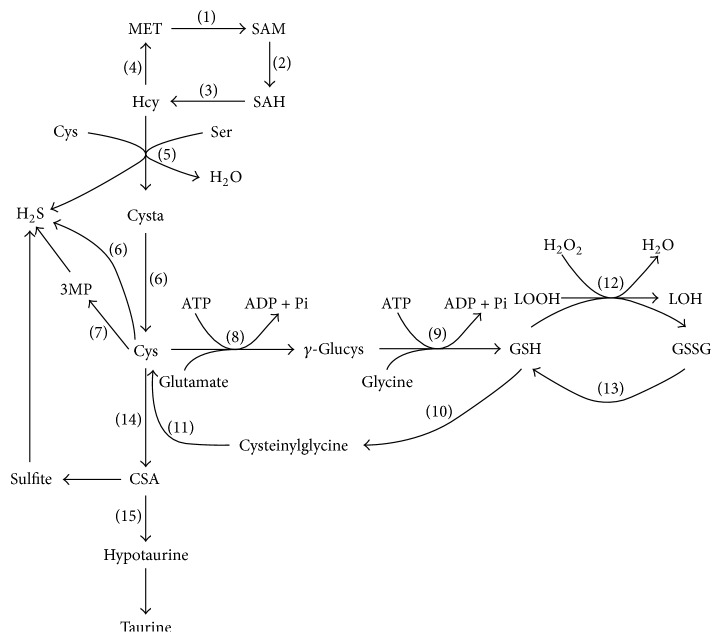
MET, methionine; Hcy, homocysteine; SAM, S-adenosylmethionine; SAH, S-adenosylhomocysteine; Ser, serine; Cys, cysteine; Cysta, cystathionine; 3MP, 3-mercaptopyruvate; H_2_S, hydrogen sulfide; *γ*-Glucys, *γ*-glutamylcysteine; GSH, glutathione; GSSG, glutathione disulfide; LOOH, lipid hydroperoxide; (1) methionine adenosyltransferase; (2) DNA methyltransferase; (3) S-adenosylhomocysteine hydrolase; (4) methionine synthase; (5) cystathionine *β*-synthase; (6) cystathionine *γ*-lyase; (7) cationic amino acid transporters; (8) *γ*-glutamylcysteine synthetase; (9) glutathione synthetase; (10) *γ*-glutamyl transpeptidase; (11) dipeptidase; (12) glutathione peroxidase; (13) glutathione reductase; (14) cysteine dioxygenase; (15) cysteine sulfinate decarboxylase.

Sulfur amino acids (SAAs) are a kind of amino acids which contain sulfhydryl. Among the SAAs, methionine and cysteine are deemed as the primary SAAs. Methionine is an indispensable amino acid in mammals as it cannot be synthesized in amounts sufficient to maintain the normal growth of mammals. Nevertheless, cysteine is a semiessential amino acid in mammals, because cysteine can be produced through the transsulfuration pathway from L-methionine degradation. Thus, the content of methionine and cysteine is considered to represent the requirement of SAAs in the diet of mammals. Increasing evidence reveals that SAAs play a crucial role in protein structure, metabolism, immunity, and oxidation [1–4]. They exert momentous functions through their metabolites, such as S-adenosylmethionine (SAM), polyamines, taurine, and glutathione (GSH) (Figure 1).

Redox homeostasis is the premise of maintaining homeostatic equilibrium of organism, and it highly depends on the balance of prooxidative and antioxidative system [5, 6]. Reactive oxygen species (ROS) is a major factor in the formation of oxidative damage, because ROS can oxidize biomolecules (including lipid, protein, and DNA) easily and thereby impairs antioxidative system and causes oxidative stress [7, 8]. Therefore, the antioxidation of SAAs attracts people's interest gradually and researchers have done many researches on it [9, 10]. A great number of researches report that SAAs have an alleviating action on various oxidant stress model, such as diabetes [11, 12], HIV infection [13], and aging [14]. Thus, our review reorganizes and highlights the antioxidation effect of two main SAAs (methionine and cysteine).

## 2. Methionine

In the protein structure, all amino acid residues are prone to be oxidized by diversified forms of ROS, especially methionine residues, as they are sensitive to almost all forms of ROS and the oxidation of methionine residues is reversible [15]. It is the main reason that methionine has the ability to resist oxidation.

### 2.1. Methionine Oxidation Reduction Cycle

**Figure 2:**
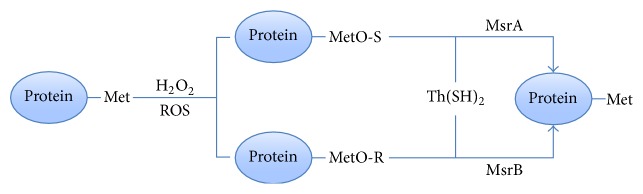
The oxidation and reduction reaction of methionine residues.

Methionine residues are extremely sensitive to ROS, and they are prone to combine with ROS and then convert to methionine sulfoxide (MetO); thereby ROS loses its activity. The reaction product of MetO is a mixture which consists of the two stereoisomers, MetO-S and MetO-R. MetO-S and MetO-R can be reduced to methionine by the thioredoxin [Th(SH)_2_] through the catalysis of methionine sulfoxide reductases A (MsrA) and methionine sulfoxide reductases B (MsrB), respectively (Figure 2). Each cycle of methionine residues oxidation and reduction will eliminate hazardous substances (e.g., hydroperoxide, hypochlorous, ozone, and lipid peroxide), which might represent a major natural scavenging system for the hazardous substances.

MrsA and MsrB are regarded as the ultimate antioxidant defence mechanisms because they are in charge of the reduction in MetO [16]. Many experiments in different objects evidenced that the level of MsrA is correlated with the elimination of the accumulated oxidative damage [17–19]. Marchetti et al. [20] proposed that the reduction of MsrA levels caused the accumulation of ROS in human lens cell. Moreover, Yermolaieva and his colleagues [21] found that the overexpression of MsrA significantly reduced the hypoxia-induced increase of ROS and maintained the normal growth of PC12 cells. MrsB has been discovered for only a short time [22], and its main function was now known to reduce oxidized MetO together with MsrA. The other functions of MsrB are remaining to the further exploration.

### 2.2. SAM

SAM is the direct product of methionine in the catalysis by methionine adenosyltransferase (MAT), and it is well known as the methyl donor for the majority of methyltransferases that modify DNA, RNA, and other proteins. SAM exerts the antioxidant capacity by this pathway: SAM increases the activity of cystathionine *γ*-synthase (CBS) which is the primary enzyme in transsulfuration and contributes to the synthesis of cysteine, thereby increasing the GSH level. Many studies show that SAM administration alleviates oxidant stress and restores the tissues. For example, Li et al. [23] found that SAM administration protects cells and inhibits oxidative stress induced by amyloid-*β*, and it activates endogenous antioxidant system by restoring the normal GSH/GSSG ratio and increasing the activities of glutathione peroxidase (GSH-Px), glutathione-S-transferase (GST), and superoxide dismutase (SOD).

### 2.3. Administration of Methionine

It is reported that the supplementation of methionine mitigated the ROS-induced damage by increasing the activity of GSH [24]. Interestingly, methionine restriction, which restricts the methionine supplementation in animal diet, is also reported to alleviate oxidant stress. For example, methionine restriction significantly reduces mitochondrial ROS generation [25, 26]. In addition, methionine deficiency in a dietary model causes a series damage to body, like hepatic pathology [27], suppression of intestinal epithelial growth [28], impairment of growth performance [29], and so on, while excessive methionine supplementation may lead to methionine poisoning and even shorten the lifespan of animals [30]. What is more, the requirement of methionine in different stages of animals is discrepant. Thus, the administration of methionine for animal production is a valuable research topic.

## 3. Cysteine

Similar to the methionine residues, cysteine residues also easily suffered from oxidation. Cysteine residues are with the properties of regulating redox since its special chemical characteristics made it easily react with H_2_O_2_ [31, 32]. In addition, serving as a precursor for GSH, cysteine is the limiting amino acid of glutathione synthesis in transsulfuration pathway. Moreover, the antioxidant property of cysteine is mainly reflected by the product of GSH, hydrogen sulfide (H_2_S), and taurine.

### 3.1. GSH

In mammals, GSH is mainly synthetized by two enzymatic ATP-dependent reactions from cysteine, glutamate, and glycine: (1) Cysteine and glutamate consume ATP to form *γ*-glutamylcysteine (*γ*-Glucys) by the catalysis of *γ*-glutamylcysteine synthetase (GCS). (2) GSH synthetase catalyzes *γ*-Glucys and glycine to form GSH, and this reaction also consumes ATP (Figure 1). In the synthesis of GSH in cell, cysteine is the rate-limiting reaction substrate [33] and supplementation with L-cysteine in humans improves synthetic rate and concentration of GSH [34]. What is more, Yin et al. [35] quantified the main source of GSH precursors by supplementation with different concentrations of L-cysteine, L-glutamate, and glycine in mice diet, and their result revealed that dietary with L-cysteine and L-glutamate increased the concentration of GSH in liver, while they also found that the excessive supplementation of L-cysteine inhibited GSH synthesis.

GSH is a cysteine-containing tripeptide and plays a vital role in cellular antioxidation in animal [36]. GSH is easily oxidized by the free radicals and other ROS (e.g., lipid peroxyl radical, H_2_O_2_, and hydroxyl radical) to form glutathione disulfide (GSSG) by the catalysis of GSH-Px. And then by the catalysis of glutathione reductase, GSSG is reduced to GSH. Therefore, cycle of GSH/GSSG contributes to the scavenging of free radicals and other reactive species and to the prevention of oxidation of biomolecules. In addition, as the substrate of GSH-Px, GSH also plays an assistant role in the antilipid peroxidation of GSH-Px. It is generally believed that the low level of GSH may lead to lipid peroxidation. For example, Agar et al. [37] employed ethanol to consume the GSH in cerebellum of mice and then found that lipid peroxidation was increased significantly. Thus, the concentration of GSH and the activities of GSH-related enzyme acted as the sign of antioxidant status in the body.

### 3.2. H_2_S

H_2_S has long been considered as a toxic gas produced in substantial amounts by mammalian tissues, while recent research reveals that it is an anti-inflammatory, antioxidant, and neuroprotective agent and plays very important roles in many physiological functions [38]. L-Cysteine is a major substrate to produce about 70% endogenous H_2_S by either enzyme (cystathionine *β*-synthase and cystathionine *γ*-lyase) [39]. And, in recent years, it is observed that D-cysteine produces H_2_S by a novel pathway and it may be more effective than L-cysteine in protecting primary cultures of cerebellar neurons from oxidative stress induced by hydrogen peroxide [40]. H_2_S is a potent antioxidant, except for directly scavenging the reactive oxygen and nitrogen species to protect tissues [41]; it also increases the activity of *γ*-glutamylcysteine synthetase and upregulates cystine transport, thereby enhancing the production of GSH to resist oxidant stress [42]. Furthermore, it is reported that H_2_S may protect gastric mucosal epithelial cells against oxidative stress through stimulation of MAP kinase pathways [43]. These pathways provide the mechanisms for H_2_S to protect the tissues from oxidative stress.

### 3.3. Taurine

Taurine is the most abundant free amino acid in mammals, and it plays an important role in many physiological functions, like visual development, neural development, detoxification, antioxidation, anti-inflammatory, and so on. Two main sources contribute to taurine synthesis in the mammals: absorption from diets and the metabolism of cysteine. Taurine is synthesized by three steps: first, cysteine is catalyzed to form cysteine sulfinate by the catalysis of cysteine dioxygenase; second, cysteine sulfinate removes carboxyl to form hypotaurine by cysteine sulfinate decarboxylase; third, hypotaurine is oxidized to taurine. Many researches confirm that increasing the dosage of cysteine in diet contributes to the activation of cysteine dioxygenase [44], and dietary supplementation of cysteine increased plasma taurine level in HIV-infection people [13].

In particular, taurine shows its protection for tissue in many models which are induced by varies oxidants [45, 46]. The antioxidant capacity of taurine is associated with ROS scavenging. Chang et al. [46] proved that taurine supplements in rat diet lowered the hyperhomocysteinemia-induced ROS production, and Palmi et al. [47] reported that taurine inhibited the production of ROS by stimulating mitochondrial Ca^2+^ absorption. In addition, taurine also increases the activities of many antioxidant enzymes in oxidant-induced models. It is confirmed that taurine restores the activities of Mn-SOD and GSH-Px in mice mitochondrion after tamoxifen infection [48]. Furthermore, Choi and Jung [49] in their studies pointed out that taurine supplementation increased hepatic SOD activity on the calcium deficiency condition, but the activities of GSH-Px and catalase (CAT) were not significantly different between normal mice and calcium deficient mice.

## 4. Conclusion

In conclusion, as the powerful antioxidants, SAAs play a curial role in maintaining the equilibrium and stability of free radicals in the body. Hence, SAAs are widely used as food additive and applied to medical care and animal breeding. Although SAAs have the excellent antioxidant capacity, of particular note is the administration of SAAs in the process of animal production, because different dosage of SAAs may have different effects on animals. Thus, further study about the appropriate dosage of SAAs will be explored in animal feeding.

## Abbreviations

SAA: Sulfur amino acids
SAM: S-Adenosylmethionine
GSH: Glutathione
ROS: Reactive oxygen species
MetO: Methionine sulfoxide
Th(SH)_2_: Thioredoxin
MsrA: Methionine sulfoxide reductase A
MsrB: Methionine sulfoxide reductase B
MAT: Methionine adenosyltransferase
CBS: Cystathionine *γ*-synthase
GSH-Px: Glutathione peroxidase
GST: Glutathione-S-transferase
SOD: Superoxide dismutase
H_2_S: Hydrogen sulfide
*γ*-Glucys: *γ*-Glutamylcysteine
GCS: *γ*-Glutamylcysteine synthetase
GSSG: Glutathione disulfide.

## References

[1] Kim S. W. (2003). *Amino Acids and Immune Function*.

[2] Grimble R. F. (2006). The effects of sulfur amino acid intake on immune function in humans. *Journal of Nutrition*.

[3] Wu G. (2009). Amino acids: metabolism, functions, and nutrition. *Amino Acids*.

[4] Brosnan J. T., Brosnan M. E. (2006). The sulfur-containing amino acids: an overview. *Journal of Nutrition*.

[5] Yin J., Ren W., Liu G. (2013). Birth oxidative stress and the development of an antioxidant system in newborn piglets. *Free Radical Research*.

[6] Duan J., Yin J., Ren W. (2016). Dietary supplementation with l-glutamate and l-aspartate alleviates oxidative stress in weaned piglets challenged with hydrogen peroxide. *Amino Acids*.

[7] Yin J., Ren W., Duan J. (2014). Dietary arginine supplementation enhances intestinal expression of SLC7A7 and SLC7A1 and ameliorates growth depression in mycotoxin-challenged pigs. *Amino Acids*.

[8] Yin J., Wu M. M., Xiao H. (2014). Development of an antioxidant system after early weaning in piglets. *Journal of Animal Science*.

[9] Martínez Y., Li X., Liu G. (2017). The role of methionine on metabolism, oxidative stress, and diseases. *Amino Acids*.

[10] Liu G., Yu L., Fang J. (2017). Methionine restriction on oxidative stress and immune response in dss-induced colitis mice. *Oncotarget*.

[11] Sekhar R. V., Mckay S. V., Patel S. G. (2011). Glutathione synthesis is diminished in patients with uncontrolled diabetes and restored by dietary supplementation with cysteine and glycine. *Diabetes Care*.

[12] Jain S. K., Velusamy T., Croad J. L., Rains J. L., Bull R. (2009). l-Cysteine supplementation lowers blood glucose, glycated hemoglobin, CRP, MCP-1, and oxidative stress and inhibits NF-*κ*B activation in the livers of Zucker diabetic rats. *Free Radical Biology & Medicine*.

[13] Borges-Santos M. D., Moreto F., Pereira P. C. M., Ming-Yu Y., Burini R. C. (2012). Plasma glutathione of HIV+ patients responded positively and differently to dietary supplementation with cysteine or glutamine. *Nutrition Journal*.

[14] Sekhar R. V., Patel S. G., Guthikonda A. P. (2011). Deficient synthesis of glutathione underlies oxidative stress in aging and can be corrected by dietary cysteine and glycine supplementation. *American Journal of Clinical Nutrition*.

[15] Stadtman E. R., Van Remmen H., Richardson A., Wehr N. B., Levine R. L. (2005). Methionine oxidation and aging. *Biochimica et Biophysica Acta (BBA) - Proteins and Proteomics*.

[16] Swennen Q., Geraert P.-A., Mercier Y. (2011). Effects of dietary protein content and 2-hydroxy-4-methylthiobutanoic acid or dl-methionine supplementation on performance and oxidative status of broiler chickens. *British Journal of Nutrition*.

[17] Prentice H. M., Moench I. A., Rickaway Z. T., Dougherty C. J., Webster K. A., Weissbach H. (2008). MsrA protects cardiac myocytes against hypoxia/reoxygenation induced cell death. *Biochemical and Biophysical Research Communications*.

[18] Lee J. W., Gordiyenko N. V., Marchetti M. (2006). Gene structure, localization and role in oxidative stress of methionine sulfoxide reductase a (MSRA) in the monkey retina. *Experimental Eye Research*.

[19] Ruan H., Tang X. D., Chen M.-L. (2002). High-quality life extension by the enzyme peptide methionine sulfoxide reductase. *Proceedings of the National Acadamy of Sciences of the United States of America*.

[20] Marchetti M. A., Lee W., Cowell T. L., Wells T. M., Weissbach H., Kantorow M. (2006). Silencing of the methionine sulfoxide reductase A gene results in loss of mitochondrial membrane potential and increased ROS production in human lens cells. *Experimental Eye Research*.

[21] Yermolaieva O., Xu R., Schinstock C. (2004). Methionine sulfoxide reductase A protects neuronal cells against brief hypoxia/reoxygenation. *Proceedings of the National Acadamy of Sciences of the United States of America*.

[22] Kim G., Weiss S. J., Levine R. L. (2014). Methionine oxidation and reduction in proteins. *Biochimica et Biophysica Acta (BBA) - General Subjects*.

[23] Li Q., Cui J., Fang C. (2017). S-Adenosylmethionine attenuates oxidative stress and neuroinflammation induced by amyloid-*β* through modulation of glutathione metabolism. *Journal of Alzheimer's Disease*.

[24] del Vesco A. P., Gasparino E., Grieser D. O. (2014). Effects of methionine supplementation on the redox state of acute heat stress-exposed quails. *Journal of Animal Science*.

[25] Caro P., Gomez J., Sanchez I. (2009). Forty percent methionine restriction decreases mitochondrial oxygen radical production and leak at complex I during forward electron flow and lowers oxidative damage to proteins and mitochondrial DNA in rat kidney and brain mitochondria. *Rejuvenation Research*.

[26] Maddineni S., Nichenametla S., Sinha R., Wilson R. P., Richie J. P. (2013). Methionine restriction affects oxidative stress and glutathione-related redox pathways in the rat. *Experimental Biology and Medicine*.

[27] Oz H. S., Chen T. S., Neuman M. (2008). Methionine deficiency and hepatic injury in a dietary steatohepatitis model. *Digestive Diseases and Sciences*.

[28] Bauchart-Thevret C., Stoll B., Chacko S., Burrin D. G. (2009). Sulfur amino acid deficiency upregulates intestinal methionine cycle activity and suppresses epithelial growth in neonatal pigs. *American Journal of Physiology-Endocrinology and Metabolism*.

[29] Wen C., Jiang X. Y., Ding L. R., Wang T., Zhou Y. M. (2017). Effects of dietary methionine on growth performance, meat quality and oxidative status of breast muscle in fast- and slow-growing broilers. *Poultry Science*.

[30] Chaturvedi P., Kamat P. K., Kalani A., Familtseva A., Tyagi S. C. (2016). High methionine diet poses cardiac threat: a molecular insight. *Journal of Cellular Physiology*.

[31] D'Autréaux B., Toledano M. B. (2007). ROS as signalling molecules: mechanisms that generate specificity in ROS homeostasis. *Nature Reviews Molecular Cell Biology*.

[32] Azad M. A. K., Huang P., Liu G. (2017). Hyperhomocysteinemia and cardiovascular disease in animal model. *Amino Acids*.

[33] Atmaca G. (2004). Antioxidant effects of sulfur-containing amino acids. *Yonsei Medical Journal*.

[34] Badaloo A., Reid M., Forrester T., Heird W. C., Jahoor F. (2002). Cysteine supplementation improves the erythrocyte glutathione synthesis rate in children with severe edematous malnutrition. *American Journal of Clinical Nutrition*.

[35] Yin J., Ren W., Yang G. (2016). l-Cysteine metabolism and its nutritional implications. *Molecular Nutrition & Food Research*.

[36] Wu G., Fang Y.-Z., Yang S., Lupton J. R., Turner N. D. (2004). Glutathione metabolism and its implications for health. *Journal of Nutrition*.

[37] Agar E., Demir Ş., Amanvermez R., Boşnak M., Ayyildiz M., Çelik C. (2000). The changes in lipid peroxidation and GSH levels in the cerebellum of rats induced by ethanol consumption are prevented by vitamin E. *Neuroscience Research Communications*.

[38] Łowicka E., Bełtowski J. (2007). Hydrogen sulfide (H2S)—the third gas of interest for pharmacologists. *Pharmacological Reports*.

[39] McBean G. J. (2012). The transsulfuration pathway: a source of cysteine for glutathione in astrocytes. *Amino Acids*.

[40] Shibuya N., Koike S., Tanaka M. (2013). A novel pathway for the production of hydrogen sulfide from D-cysteine in mammalian cells. *Nature Communications*.

[41] Szabó C. (2007). Hydrogen sulphide and its therapeutic potential. *Nature Reviews Drug Discovery*.

[42] Kimura Y., Goto Y.-I., Kimura H. (2010). Hydrogen sulfide increases glutathione production and suppresses oxidative stress in mitochondria. *Antioxidants & Redox Signaling*.

[43] Yonezawa D., Sekiguchi F., Miyamoto M. (2007). A protective role of hydrogen sulfide against oxidative stress in rat gastric mucosal epithelium. *Toxicology*.

[44] Stipanuk M. H. (2004). Sulfur amino acid metabolism: pathways for production and removal of homocysteine and cysteine. *Annual Review of Nutrition*.

[45] Sinha M., Manna P., Sil P. C. (2007). Taurine, a conditionally essential amino acid, ameliorates arsenic-induced cytotoxicity in murine hepatocytes. *Toxicology in Vitro*.

[46] Chang L., Xu J., Yu F., Zhao J., Tang X., Tang C. (2004). Taurine protected myocardial mitochondria injury induced by hyperhomocysteinemia in rats. *Amino Acids*.

[47] Palmi M., Davey G., Tipton K. F., Meini A. (2006). Taurine, taurine analogues, and mitochondrial function and dysfuntion. *Advances in Experimental Medicine and Biology*.

[48] Parvez S., Tabassum H., Banerjee B. D., Raisuddin S. (2008). Taurine prevents tamoxifen-induced mitochondrial oxidative damage in mice. *Basic & Clinical Pharmacology & Toxicology*.

[49] Choi M.-J., Jung Y.-J., Lee D.-H., Schaffer S. W., Park E., Kim H. W. (2017). Effects of taurine and vitamin D on antioxidant enzyme activity and lipids profiles in rats fed diet deficient calcium. *Taurine 10*.

